# Comfort food concepts and contexts in which they are used: A scoping review protocol

**DOI:** 10.1371/journal.pone.0299991

**Published:** 2024-04-24

**Authors:** Juliana Miranda Pereira, Rute Guedes Melo, Joyanne de Souza Medeiros, Anna Cecília Queiroz de Medeiros, Fívia de Araújo Lopes, on behalf of Federal University of Rio Grande do Norte

**Affiliations:** 1 Department of Psycholobiology, Federal University of Rio Grande do Norte, Natal, Rio Grande do Norte, Brazil; 2 Faculdade de Ciências da Saúde, Federal University of Rio Grande do Norte, Santa Cruz, Rio Grande do Norte, Brazil; FMUP: Universidade do Porto Faculdade de Medicina, PORTUGAL

## Abstract

**Objective:**

The objectives of this study are to clarify the scientific definition of comfort food, identify which methodologies are being used in research on this topic and which factors are associated with the consumption of comfort food.

**Introduction:**

The consumption of comfort foods is subjective and influenced by individual experiences, as they are known and appreciated by the person. However, divergences about the definition of comfort food in the scientific literature reflect the heterogeneity of the methods used in the research, and consequently identification of possible factors associated with the consumption of this type of food, which can influence the knowledge about the consumption of these foods and their potential effects on the health of those who consume them.

**Inclusion criteria:**

Works with a qualitative and quantitative approach published in full in indexed sources or in gray literature, available online in the databases consulted, without restriction on language or year of publication will be included.

**Methods:**

The protocol was built based on the methodological recommendations of the Joanna Briggs Institute (JBI) for scoping reviews and the recommendations of the Preferred Reporting Items for Systematic reviews and Meta-Analyses extension for Scoping Reviews (PRISMA-ScR). The Participants, Concept and Context (PCC) mnemonic strategy was built: general population, comfort food concept and world context. Based on this, search strategies were developed for different databases. Instruments were also developed for recording documents, extracting data, justifying the exclusion of documents and not obtaining access to content. A Pilot Study was conducted to test the developed methodology and instruments. The protocol has been registered with the Open Science Framework (OSF) (https://osf.io/gnza4/). The results will be presented in the review resulting from this protocol in three ways: accounting of the documents will be recorded in a PRISMA Flow Diagram, the main information of the studies and their frequencies will be presented in a table, and the union of these outcomes will be presented visually in a Graphical Abstract.

## Introduction

The food history of our ancestors within the context of scarcity, climate change, need for reproduction, as well as our own and our offspring’s survival, shaped the nutritional needs of modern humans. As the environment changed due to climate change, our ancestors adapted their food consumption [1,2]. One adaptation resulting from the evolutionary process is the strong taste preference and appreciation we have for fat and sugar, because their dietary sources such as ripe fruits and meats were scarce in the past [3].

The behaviors of searching for food (craving) and food consumption are reinforced by reward pathways in the brain as they are determinant components of human survival, which, integrated with nutritional status and sensory information, constitute the rewarding properties and affective value of food, culminating in appetite and individual eating behavior, including the pleasurable response of appreciation (liking) [4]; in addition, personality and cognitive characteristics can influence physiological changes induced by food and influence food-related memory. The already lived sensory experiences of a food contribute to the emotional weight of food situations and are related to memories. However, it is the emotions related to the situations, such as those experienced after the meal and the emotional context of the meal situation—for example, a celebration—that trigger spontaneous memories [5,6].

According to Locher et al. [7], the definition of comfort food was added in 1997 to two renowned dictionaries: the Collegiate Dictionary, which defined it as: “food prepared in a traditional style with a generally nostalgic or sentimental appeal”; and the Oxford dictionary, which defined the term as: “food that comforts or provides consolation; therefore, any food (often high in sugar or carbohydrate) that is associated with childhood or home cooking” [7]. The definition currently available on the Oxford Dictionary website is “foods that make you feel better, generally because they contain too much sugar or because they remind you of home” [8]. Furthermore, the term comfort food has often been used in common sense by popular media, such as food and cooking TV shows and magazines, to represent a high calorie food set [9].

Unlike what currently appears to be in public knowledge, consensus on the definition of comfort food in academic literature is difficult [9]. Two widely referenced studies in this research area were developed in 2003. Dallman et al. [10] define these foods as “palatable foods, whose sensory qualities indicate calories”. Wansink, Cheney and Chan [11] state that comfort foods are “foods that promote physiological and psychological comfort when consumed”, diverging from the previous definition.

Despite the divergence, more research was carried out on the subject after these definitions, but without defining which concept is used to guide the study development. However, it is common to use or question the consumption of hyperpalatable foods as comfort foods in their research methods, such as sugary drinks, snacks, fast foods and sweets [12–17]. On the other hand, some studies present a definition, such as Locher et al. [7] who defined it as “food that makes you feel good or promotes comfort”. Still, there are studies which have asked the participants to present their own definition of comfort food [9], reflecting the heterogeneity of the concept in the methods employed, and consequently in identification of possible factors associated with the consumption of this type of food, which are still not well elucidated in the scientific literature and can be influenced by the concept and methodology employed.

Reviews on the subject were developed [18,19] and one of them conceptualized comfort food as “those foods whose consumption provides comfort or a feeling of well-being” [19]. However, these studies did not follow search methodologies, selection or systematic analysis of scientific evidence that guarantee the rigor and coherence of the information.

It is known that comfort foods are foods already known and appreciated by the individual [11], characterizing the consumption of these foods as something subjective and influenced by individual experiences. Therefore, any food that is appreciated by the individual and that has a related affective memory can be a comfort food [7]. Likewise, “palatable calorie-reporting” foods may not be considered comfort foods for some people. In addition, it is important to consider that palatable foods are not only consumed in the context of seeking “physiological and psychological comfort”.

Therefore, categorizing comfort foods as palatable foods that suggest calories, especially ultra-processed ones, can limit knowledge about the consumption of these foods and their potential effects on the health of those who consume them. Considering the complexity of the subject, carrying out a scoping review to clarify this concept, as well as mapping the methodologies used and the associated factors is important to inform researchers about the applicability of the term, support decision-making for subsequent studies, and consequently promote progress in this knowledge area in an improved way.

A previous search was performed in JBI Evidence Synthesis, Figshare, Open Science Framework (OSF), PubMed and Cumulative Index to Nursing and Allied Health Literature (CINAHL) databases and no published scoping or systematic review on the topic or currently ongoing was identified. Therefore, objectives of this study are to clarify the scientific definition of comfort food considering how it is being understood, in what context and how it is being used; and identify which methodologies are being used in research on this topic and the factors associated with the consumption of comfort food based on available scientific documents.

## Methods

The methodology proposed by the Joanna Briggs Institute (JBI) [20] for scoping reviews and the recommendations of the Preferred Reporting Items for Systematic reviews and Meta-Analyses extension for Scoping Reviews (PRISMA- ScR) [21] were followed in elaborating this scoping review protocol. The protocol has been registered with the Open Science Framework (OSF) (https://osf.io/gnza4/). Also, this article was written based on the Prisma-P Checklist (2015) guidelines and is available on the OSF page for this protocol (https://osf.io/jy876). Given the dynamism inherent in the research process, it is possible that the methodology will undergo adjustments. Therefore, if they occur, such changes will be reported and detailed in the final scoping review in order to ensure that the decisions taken were based on solid scientific criteria, transparent and consistent with the research objectives.

## Review questions

The study questions were formulated using the Participants, Concept and Context (PCC) mnemonic strategy. In this protocol, participants consist of the general population, including all age groups and social characteristics; the concept to be explored is that of comfort food; and the context is global. Thus, the guiding question of this scoping review is: How is comfort food defined and how is its concept used in research in the areas of food, nutrition and eating behavior? And the complementary questions: What methodologies are used in studies on comfort food? What are the factors associated with the consumption of comfort food?

### Inclusion and exclusion criteria

Works with a qualitative and quantitative approach published in full in indexed sources or in gray literature, available online in the consulted databases, without restriction on language or year of publication will be included. Research reports, editorials, letters to the editor and abstracts published in conference proceedings, reports or case series, communications and duplicate documents will be excluded. The databases to be consulted will be: Medline/PubMed; Embase; Cochrane Library Databanks; Lilacs; Cumulative Index to Nursing and Allied Health Literature (CINAHL); Scopus; Scielo; Web of science; Science direct; Directory of Open Access Journals (DOAJ); Directory of Open Access Books (DOAB). The following sites will be accessed to search the gray literature: CAPES Catalog of Theses and Dissertations; DART-Europe E-theses Portal; Electronic Theses Online Service (EThOS); Academic Archive Online (DIVA); Brazilian Digital Library of Theses and Dissertations (BDTD); Cyberthesis; Google Scholar. The complete list of databases is available in Table 1. Access to the databases was through the Journal Portal of the Coordination for the Improvement of Higher Education Personnel (Capes) in the period of August 2023, but for each database there will be a specific search strategy and these will be available as complementary information to the final review.

**Table 1:** Databases selected for the study.

	Databases selected for the study
**For the published literature:**	Medline/PubMed; Embase; Cochrane Library Databanks; Lilacs; Cumulative Index to Nursing and Allied Health Literature (CINAHL); Scopus; Scielo; Web of science; Science direct; Directory of Open Access Journals (DOAJ); Directory of Open Access Books (DOAB).
**For the Gray Literature (dissertations and theses):**	CAPES Catalog of Theses and Dissertations; DART-Europe E-theses Portal; Electronic Theses Online Service (EThOS); Academic Archive Online (DIVA); Brazilian Digital Library of Theses and Dissertations (BDTD); Cybertesis; Google Scholar.

### Search strategy

An initial search was performed in January 2023 in the PUBMED database using the term “comfort food”. Articles that presented the term were selected, and then the title, abstract and keywords were analyzed to identify used index terms which could contribute to the construction of the search strategy for the review.

Search strategies were developed by a professional librarian and were peer-reviewed. Controlled vocabularies in health sciences were used to retrieve relevant studies in national and international multidisciplinary databases and in the health area, namely: Medical Subject Headings (MeSH), Health Sciences Descriptors (DeCS) and Emtree (Embase), with the purpose of identifying descriptors to broaden the search results. In addition, keywords were used. Some of the descriptors and keywords to be used include: “Comfort food”; “Comfort eating”; “Food preferences”; and “Emotional eating”, as detailed in Table 2.

**Table 2:** PubMed search strategy.

#	Searches
1	“Food Preferences"[Mh] OR “Food Preferences”[tiab] OR “Food Selection*”[tiab] OR “Feeding Behavior”[Mh] OR “Feeding Behavior*”[tiab] OR “Feeding Related Behavior*”[tiab] OR “Feeding-Related Behaviors”[tiab] OR “Reward-related eating”[tiab] OR “Food Habit*”[tiab] OR “eating habit*”[tiab] OR “eating behavior”[tiab]
2	“stress eating”[tiab] OR Belongingness[tiab] OR Attachment[tiab] OR satisfaction[tiab] OR Tastiness[tiab] OR “food intake”[tiab] OR “Food liking”[tiab] OR “food choices”[tiab]
3	#1 AND #2 16,868 records Concept A
4	“Comfort food*”[tiab] OR “comfort eating”[tiab] OR “emotional eating”[tiab] OR "palatable food"[tiab]
5	#4 Concept B
6	#3 AND #5 484 records This line combines A AND B

It is noteworthy that as greater familiarization with the databases occurs, more keywords, sources and other descriptors can be discovered and incorporated into the search strategy taking into account the interactive nature of this study. Therefore, it is reiterated that if changes occur, these will be incorporated and reported in the review. In addition, reference lists of materials included in the review will be consulted in order to identify additional sources.

### Data extraction

Searches in the databases will follow an order of each database at a time, and will be carried out by one of the reviewers in an anonymous browser. Duplicates will be excluded from the results. Then, all remaining publications from the search will be extracted and transferred to Rayyan, a free and intuitive application available via website and mobile, which aims to assist in screening eligible studies for systematic reviews [22]. The initial screening will occur independently between two reviewers by reading the title and abstract of the material, keeping those that contain information relevant to the research objectives. The selection process will follow the recommendations contained in the PRISMA-ScR checklist and will have an expected deadline of early November 2023. All extracted articles will be accounted for in Table 3. If the articles only refer to comfort food but a concept has not been attributed by the authors or a reference is made to a concept by other authors, they will be included.

**Table 3:** Document accounting.

Data	Accessed database	Search results (number)	Excluded	Total
				
				
				
				

The second screening begins with a complete reading of the selected articles from which only data related to the PCC will be extracted, such as the methodology used, the concept of comfort food, in which context it was used and which factors are associated with the consumption of comfort food. These data will be recorded in Table 4, constructed by the work team based on the recommendations of Peters et al. [23]. Considering that there may be disagreements at some article selection stage, these will be resolved in discussion with a third reviewer who will make the decision. The agreement level between the reviewers and the facilitator will be calculated and reported. In addition, how the disagreement was resolved will also be informed in the final review report.

**Table 4:** Data extraction.

**Reference**					
**Year**					
**Title**					
**Study design/type**					
**Origin/country**					
**Objective**					
Population**	**Sample number**				
	**Age range**				
	**Biological sex**				
	Group*** (i.e. university students)				
**Methodology / methods**					
Type of procedure (i.e. intervention duration)**					
Instruments/assessments used****		Insert whether scales and tests were used, and if so, which ones.			
Results and details**					
**Key findings related to the scoping review question(s).**	**Concept of comfort food**	How is comfort (food) defined in the study.			
	**Reference of the concept**	What references are used in the article to refer to the concept of comfort (food).			
	Context in which it was used*	Specify if the article was used in the context of nutrition, or psychology.			
	**Factors associated with comfort food consumption**	Insert which aspects/elements are related to the concept of comfort food.			

It is shown that the articles not included in the review will be registered in the exclusion justification table (Table 5). The authors of works not available in full will be contacted in order to gain access to the content. If this is not available, the title of the study and the reason for not obtaining the data will be recorded in a table (Table 6).

**Table 5:** Justification for document exclusion.

Reference	Year	Title	Reason for excluding the full article
			
			
			
			

**Table 6:** Not gaining access to content.

Reference	Title	Reason for contact	Type of contact	Contact Method	Attempts (max. 3)
					
					
					
					

A Pilot Study was conducted following the steps mentioned above in order to assess the adequacy of the search strategy, methodology and instruments to be used in the review. A search was performed on PubMed (using the search strategy available in Table 2) and the first 50 resulting articles were selected. The three reviewers separately read the titles and abstracts and selected the articles. After that, there was a meeting to share the selection results, analyze divergences and align decisions. Then, the reviewers separately filled in the data extraction table with the information contained in the selected articles. At the end, improvements were discussed and put in the table, divergences were resolved and the way of tabulating the extracted data was aligned.

From the 50 articles extracted, the title and abstract were analyzed, considering the recommended inclusion and exclusion criteria. Of these, three met the criteria. Then, the full reading documents phase began. After that, the data extraction table (Table 4) was complete. During this process, some improvement needs were identified, such as: adding the "type of study" and reorganizing some columns to make data identification and filling faster: the positions of "age group" and "sample number" were reversed (this one now comes first) and "group" with biological sex (this one comes after). The other columns remained as in the original table. The pilot study results are available in Table 7 and on the OSF page for this protocol (https://osf.io/sxm85).

**Table 7:** Pilot study results.

**Reference**		McKay, N., Przybysz, J., Cavanaugh, A., Horvatits, E., Giorgianni, N., & Czajka, K. (2021). The effect of unhealthy food and liking on stress reactivity. Physiology & Behavior, 229, 113216. https://doi.org/10.1016/j.physbeh.2020.113216	Kaiser, B., Gemesi, K., Holzmann, S. L., Wintergerst, M., Lurz, M., Hauner, H., Groh, G., Böhm, M., Krcmar, H., Holzapfel, C., & Gedrich, K. (2022). Stress-induced hyperphagia: empirical characterization of stress-ovaereaters. BMC Public Health, 22(1). https://doi.org/10.1186/s12889-021-12488	Finkelstein-Fox, L., Gnall, K. E., & Park, C. L. (2020). Mindfulness moderates daily stress and comfort food snacking linkages: a multilevel examination. Journal of Behavioral Medicine, 43(6), 1062–1068. https://doi.org/10.1007/s10865-020-00164-z
**Year**		2020	2022	2020
**Title**		The effect of unhealthy food and liking on stress reactivity.	Stress-induced hyperphagia: empirical characterization of stress-ovaereaters.	Mindfulness moderates daily stress and comfort food snacking linkages: a multilevel examination.
**Origin/country**		EUA, NY.	Germany.	EUA, Connecticut.
**Study design/type**		Cross-sectional.	Cross-sectional.	Cross-sectional.
**Objective**		Investigate whether both unhealthy and healthy food items have the potential to decrease stress reactivity. Additionally, expand upon prior research by exploring whether participants’ preferences play a role in any potential stress reduction effects.	To identify the characteristics in adults in Germany related to hyperphagia under stress, focusing on perceived stress, coping, food motivation, comfort foods and personality types.	Explore the multilevel associations between daily stress evaluations and the consumption of comfort foods among undergraduate participants.
**Population**	**Age range**	• First experiment: 18–49 (m = 21). • Second experiment: 18–37 (m = 21).	Adults 18–82 years (m = 31.5, sd = 12.8).	Young adults (m = 18.9).
	**Biological sex**	Male (19,6%), female (77,4%) and unindentified (0,05%).	Male (19,2%) and female (80.8%).	Male (25,7%) and female (74,3%).
	**Group**	• First experiment participants were from Psychology courses. • Second were recruited from Psychology courses at SUNY Buffalo State.	Adults from Germany.	Undergraduate psychology students.
	**Sample number**	• First experiment:172 participants. • Second experiment: 66.	1222	261
**Methodology / methods**		• First experiment:, participants assessed baseline anxiety levels, were randomly allocated to either consume no food, eat carrots, or have a candy bar, reevaluated their anxiety levels, and provided feedback about their liking of the assigned condition. • Second experiment replicated the same protocol, but participants were exposed to a stressor before consuming a food item. Moreover, physiological stress indicators such as salivary cortisol, α-amylase, and cardiovascular measures were measured during this experiment.	Pre-tested questionnaires application on an online platform.	Participants were tasked with maintaining a diary to explore multilevel connections between daily stress assessments and the consumption of comfort foods.
**Type of procedure**		In the first experiment, conducted during laboratory visits between 10:00 a.m. and 5:00 p.m., participants were instructed to refrain from eating or drinking (except water) for 2 hours prior to the visit. Phones were removed during the session. Measurements of height and weight were taken, and participants assessed their hunger and anxiety. They were randomly assigned to one of four conditions: high energy-dense food (Twix® candy bar; n = 48), low-energy dense food (baby carrots; n = 46), a control group with no food but provided with magazines to read (to control for engaging in an activity; n = 38), or a control group with no food, sitting quietly with no magazines (n = 40). Participants had 5 minutes to complete their assigned food condition, and if provided with food, were instructed to take the full 5 minutes to eat. Following the food consumption, participants rated their liking of the condition, anxiety, hunger, eating patterns, and completed a demographic questionnaire. In the second experiment, participant visits occurred between 12:00 pm and 3:00 pm. Before the visit, participants abstained from eating or drinking (except water) for 2 hours. The procedure began with a 15-minute period where participants rinsed their mouths with water and sat quietly. Baseline measures, including cardiovascular function, anxiety, hunger, and a saliva sample (later analyzed for cortisol and α-amylase), were obtained at 0 minutes. Participants then underwent the Trier Social Stress Test (TSST), simulating acute stress through a mock job interview. Blood pressure and heart rate were measured at 5 minutes, and participants had a 5-minute anticipatory period to prepare their speech. Subsequently, they gave a 5-minute oral presentation, and measurements were taken at 10 and 15 minutes. Following the stressor, participants completed a serial subtraction task for 5 minutes. After the stressor, participants were randomly assigned to one of three food conditions: Twix® candy bar (n = 23), an equal portion of baby carrots (50.7 g; n = 26), or no food (n = 24). They had 5 minutes to eat (or read magazines if in the no food condition). Participants rinsed their mouths with water, rated their liking of their condition and their anxiety at 25 minutes. Blood pressure, heart rate, and a third saliva sample were collected at 30 minutes. Over the next 30 minutes, participants completed assessments of hunger, a demographic questionnaire, TFEQ, EAT, and read magazines. Blood pressure, heart rate, and saliva samples were collected at 40, 50, and 60 minutes.	3-month online cross-sectional study.	Participants were instructed to engage in nightly expressive writing sessions lasting approximately 5–10 minutes. Emails containing a link to the diary entry were dispatched at 8:00 PM for participants’ convenience. The diary prompts encompassed various scales, and participants diligently maintained this practice for 2560 days.
**Instruments/assessments used**		Profile of Mood States (POMS): Used to assess state anxiety. Liking of the Condition: Assessed using a 100 mm visual analog scale. Hunger: Determined through a visual analog scale featuring a 100 mm horizontal line anchored with “not at all” and “the most possible.” Relationship with Food: Assessed using the Three Factor Eating Questionnaire (TFEQ) and the Eating Attitudes Test (EAT). Demographic Questionnaire: Included to determine personal characteristics such as age, gender, race, and ethnicity. Salivary Samples: Collected using a 1.5 ml microcentrifuge tube with a salivary collection aid mouthpiece. Cortisol Measurement: Conducted with a salivary cortisol enzyme immunoassay kit, following the assay protocol. Blood Pressure and Heart Rate: Measured using an automatic digital blood pressure monitor.	• Eating stress: Salzburg Stress Eating Scale (SSES) • Perceived stress: Perceived Stress Scale (PSS) • Stress coping: Stress and Coping Inventory (SCI) • Eating motives: The Eating Motivation Survey (TEMS) • Frequency of comfort food consumption: participants were presented with a closed list of 13 pre-selected comfort foods [chocolate and chocolate. confectionery, sweets, ice cream, cakes, cookies, snacks and snacks. cookies, salted nuts, fried foods and snacks. French fries, fast food (hamburgers, curry sausages or pizza), alcohol, sugary drinks, energy drinks and coffee] and asked to indicate how often they consumed them in stressful situations. 5-point Likert scale ranging from 0 = never to 4 = very often. The answers ’often’ and ’very’ often (3 and 4) were considered to be a positive value for the consumption of comfort food and were therefore simultaneously coded with 1. The answers ’sometimes’, ’rarely’ and ’never’ were simultaneously coded with 0. • Personality categorization: Big Five Inventory (BFI-10) Anthropometric data: gender, age, weight, self-reported height".	Were measured on the diary: • Stress assessment • For comfort food intake were used 5 items from the National Cancer Institute Dietary Screener Questionnaire, measuring the frequency of sweet and salty snack food intake were assessed • Trait mindfulness was assessed using the 12-item Cognitive Affective Mindfulness Scale (CAMS-R).
**Results and details**		In both experiments, the presence of food did not yield any discernible impact on emotional or physiological stress measures. Individuals who expressed a strong preference for their assigned condition demonstrated a reduction in anxiety across both experiments and displayed improved post-stress recovery of α-amylase. The anxiolytic effects associated with liking were consistent across participants, irrespective of whether they engaged in the healthy, unhealthy, or no-food conditions.	Female participants had a higher mean SSES score compared to male participants. ‘Agreeableness’ (BigFive) was found to be a negative predictor of stress-overeating. The most pronounced difference in eating motives (The Eating Motivation Survey, TEMS) was found for ‘Affect Regulation’ and ‘Weight Control’. Participants selected (multiple) comfort foods • stress-overeaters selected 3.2 out items • stress-undereaters choose a mean of 1.7 • stress-insensitive eaters a mean of 1.5 • stress-overeaters chose chocolate & confectionery as comfort food, followed by coffee, and cookies stress coping as well as dieting status were shown to have effects on the consumption of comfort foods.	Mindfulness moderated the observed effects, indicating a negative correlation between within-person stress and comfort food consumption specifically among individuals with higher mindfulness levels. These results highlight distinct associations between chronic stress exposure and acute stress reactivity with eating behavior. The findings suggest that mindfulness and chronic stress could be essential intervention targets for non-clinical populations at risk of engaging in unhealthy eating habits.
**Key findings related to the scoping review question(s).**	**Concept of comfort food**	Comfort food is defined as a synonym of unhealthy food. "It temper mood and enhances performance on ’belongingness’ tasks when compared to consuming the same food without considering it as comfort food. However, consuming self-identified comfort foods does not temper a laboratory-induced negative mood over either an equally well liked (but not considered comforting) food item or no food."	synonyms of Hyperpalatable foods.	It tends to be consumed by people with the emotional eating style trait. It also acts as a mood regulator in stressful situations.
	**Reference of the concept**	Does not present reference.	Does not present reference.	O’Connor DB, Jones F, Conner M, McMillan B, Ferguson E. Effects of daily hassles and eating style on eating behavior. Health Psychology. 2008;27(1, Suppl):S20–31. https://doi.org/10.1037/0278-6133.27.1.S20
	Context in which it was used*	Stressful situations.	Stressful situations.	Stressful situations.
	**Factors associated with comfort food consumption**	It was not analyzed.	It was not analyzed.	• Stress evaluation was significantly associated with comfort food consumption at between-person levels, but not within-person levels • Within-person stress was negatively associated with comfort food intake for individuals with high trait mindfulness and decreased comfort food intake • The effects of internal stress were significantly negatively associated with comfort food intake for those whose CAMS-R scores were greater than 34 • Participants reported eating significantly fewer comfort foods throughout the study.

### Analysis and presentation of the results

The article accounting will be registered in the PRISMA Flow Diagram, which presents the different review stages involving the mapping of the records found, articles which were included and excluded, as well as the reason for the exclusions [24].

The main information of the studies, such as concept, application, associated factors and methodology used and their frequencies will be presented in a table. In addition, a Graphical Abstract will be created to unite and express these outcomes visually, enabling quick and easy understanding of the facts. In this context, such creation will take place through the “Mind The Graph” website, which consists of a tool with design resources and a wide collection of scientific illustrations [25]. All demonstrations of results will be accompanied by a narrative summary that will describe how the results and/or graphs relate to the questions developed and the objectives of the review. Finally, the underlying data for the results will be presented in a table, which will serve as supplementary material.

## Supporting information

S1 File Checklist PRISMA for systematic review protocols. (DOCX)
